# Population-Based Analysis of Vaccination Status and Post-Vaccination Adverse Events in Adults Aged 55 and Older

**DOI:** 10.3390/jcm14124297

**Published:** 2025-06-17

**Authors:** Adrianna Frydrysiak-Brzozowska, Beata Haor, Agnieszka Pluta, Mariola Głowacka

**Affiliations:** 1 Nursing Department, Faculty of Health Science, Collegium Medicum, The Mazovian University in Plock, 04-920 Płock, Poland; 2 Neurological and Neurosurgical Nursing Department, Faculty of Health Science, Collegium Medicum in Bydgoszcz, Nicolaus Copernicus University in Toruń, 85-094 Torun, Poland; beata.haor@pans.wloclawek.pl; 3 Department of Preventive Nursing, Faculty of Health Sciences, Collegium Medicum in Bydgoszcz, Nicolaus Copernicus University in Toruń, 85-094 Torun, Poland; agnieszka.pluta@cm.umk.pl

**Keywords:** older adults, vaccination coverage, adverse events following immunization, gender differences

## Abstract

**Background/Objectives**: Older adults face increased vulnerability to infectious diseases such as influenza, pneumococcal infections, and COVID-19. Vaccination remains a key public health strategy, yet coverage and adverse event data in this group are limited. This study aimed to assess the prevalence of vaccination and the occurrence of post-vaccination adverse events among individuals aged 55 and older in Płock, Poland, with particular attention to gender and age differences. **Methods**: A population-based cross-sectional study was conducted between January and November 2022 among 2040 adults aged ≥ 55 years. Participants completed a structured questionnaire on vaccination history (past 3 years) and adverse events following immunization (AEFI). Cognitive eligibility was assessed using the MMSE (≥27). Statistical analyses included *t*-tests, chi-square tests, and Pearson correlation coefficients with a significance level of *p* < 0.05. **Results**: COVID-19 vaccination was reported by 86.9% of participants, influenza vaccination by 45.5%, and pneumococcal vaccination by 15.1%. Women reported significantly more adverse events following COVID-19 vaccination compared to men (16.1% vs. 8.8%, *p* < 0.001). A weak negative correlation was observed between age and number of vaccinations received (r = −0.088, *p* = 0.001), while age was positively correlated with adverse events following COVID-19 vaccination (r = 0.175, *p* < 0.001). Influenza vaccination was more common among men than women (50.7% vs. 43.4%, *p* < 0.05). **Conclusions**: Vaccination coverage among older adults in Płock was highest for COVID-19 but remained suboptimal for influenza and pneumococcal vaccines. Women reported adverse events more frequently than men. These findings highlight the need for targeted vaccination strategies and gender-sensitive communication approaches.

## 1. Introduction

Vaccination remains one of the most effective public health interventions for preventing infectious diseases, particularly among older adults who face increased vulnerability due to immunosenescence and a higher prevalence of chronic conditions [1,2]. Despite strong evidence supporting vaccination benefits in this population, immunization coverage rates often fall below recommended targets for several important vaccines globally [3]. The World Health Organization (WHO) has identified vaccine hesitancy as one of the top 10 threats to global health, with particular concerns regarding older adult populations in both developed and developing nations [4,5]. Annual influenza epidemics are associated with substantial morbidity and mortality among seniors, with an estimated 70–90% of influenza-related deaths occurring in people over 65 years of age globally [6,7]. The European Centre for Disease Prevention and Control (ECDC) reports that despite recommendations, influenza vaccination coverage among older adults varies widely across European countries, ranging from below 10% to over 70% [8]. Similarly, Streptococcus pneumoniae infections remain a leading cause of community-acquired pneumonia, meningitis, and bacteremia in older adults internationally, with case fatality rates reaching 30% in those aged 65 and above [9,10]. The World Health Organization estimates that pneumococcal diseases cause approximately 1.6 million deaths annually worldwide, with disproportionate impacts on older populations [11]. The recent COVID-19 pandemic further highlighted the age-dependent vulnerability to respiratory infections, with older adults experiencing significantly higher hospitalization and mortality rates across all continents [12,13,14]. International data from organizations such as the Centers for Disease Control and Prevention (CDC) in the United States, the National Health Service (NHS) in the United Kingdom, and similar institutions in Australia, Japan, and Brazil have consistently demonstrated the effectiveness of vaccination in reducing severe COVID-19 outcomes among older adults [15,16].

In addition to the clinical burden, social and psychological determinants play a key role in shaping vaccine uptake in older age groups. Vaccination hesitancy among older adults stems from various factors that transcend national boundaries, including concerns about vaccine safety, misconceptions about efficacy, and fear of adverse events following immunization (AEFI) [17,18]. Research from multinational cohorts conducted by institutions such as the International Federation on Ageing (IFA) and the International Association of Gerontology and Geriatrics (IAGG) has identified universal barriers to vaccination uptake in this demographic [19]. Gender differences in vaccine acceptance and reporting of adverse events have been documented in previous studies across diverse cultural contexts [20,21], suggesting the need for gender-sensitive approaches to vaccination programs globally. Additionally, age-specific variations within the older adult population may influence vaccination patterns and experiences of adverse events, necessitating age-stratified analyses to inform targeted interventions [22].

In Poland, as in many countries with aging populations, such as Italy, Germany, and Japan, national vaccination recommendations include annual influenza vaccination for all individuals aged 55 and older, pneumococcal vaccination for those with specific risk factors or aged 65 and above, and COVID-19 vaccination as part of the pandemic response strategy [23,24]. However, comprehensive data on the actual uptake of these vaccines among seniors, particularly at the local level, remain limited in many regions worldwide [25]. Furthermore, real-world data on the prevalence and patterns of adverse events following immunization in this population are scarce across international settings, despite their importance for addressing vaccine hesitancy concerns [26,27].

The city of Płock, with its diverse senior population and established healthcare infrastructure, provides an important case study that can contribute to the broader international understanding of vaccination patterns among older adults. Similar studies have been conducted in urban centers across Europe (Barcelona, Spain; Copenhagen, Denmark), North America (Boston, USA; Montreal, Canada), and Asia (Kyoto, Japan; Seoul, South Korea), enabling valuable cross-cultural comparisons [28,29]. Understanding local vaccination coverage and the distribution of adverse events can inform not only municipal health policies but also contribute to global knowledge exchange through organizations, such as the International Association of Immunologists (IAI) and the Global Alliance for Vaccines and Immunization (GAVI) [30]. Additionally, the study sought to examine the occurrence of adverse events following immunization and identify potential correlates of vaccination behavior and adverse reactions in this population. Findings from this research may have implications for improving vaccination strategies for older adults across diverse international contexts and healthcare systems [31].

Therefore, the aim of this population-based study was to examine vaccination coverage and the occurrence of adverse events following immunization among adults aged 55 and older residing in Płock, Poland, with particular focus on differences by age and gender.

## 2. Materials and Methods

### 2.1. Study Design and Participants

This study is part of the research project entitled “Adherence as the responsibility of pre-seniors and seniors in the therapeutic process”, focusing on the health behaviors and attitudes of individuals aged 55 years and older. The research was conducted between January and November 2022 among participants affiliated with primary healthcare centers and Universities of the Third Age in Płock, Poland. Participants were selected based on their age (≥55 years) and residence or official registration in the city of Płock. According to data from Statistics Poland, in 2022, individuals of post-working age (women aged 60 and over and men aged 65 and over) constituted 26.4% of Płock’s population [32]. Although our study included participants aged 55 and over, this statistic helps to contextualize the demographic ageing of the local population and underscores the relevance of examining this age group. Cognitive functioning was screened using the Mini Mental State Examination (MMSE), with only individuals scoring between 27 and 30 points included, ensuring the absence of significant cognitive impairments [33,34]. Of the 2253 individuals who initially consented, 2102 met the cognitive inclusion criterion (MMSE 27–30) and proceeded to the next stage. A total of 151 people (6.7%) were excluded due to lower scores, which could have compromised the reliability of self-reported data and completion of psychometric tools. After obtaining voluntary informed consent, participants completed a structured survey either in paper format or electronically through the LimeSurvey platform (LimeSurvey GmbH, Hamburg, Germany). Out of 2253 initially consenting individuals, 2040 completed the full survey correctly and were included in the final analysis. Women comprised 68.9% (n = 1406) of the respondents. The sample was intended to mirror the demographic structure of the senior population in Płock.

### 2.2. Measures

The participants answered questions specifically regarding their vaccination history over the past 3 years. The survey captured information about vaccinations against influenza, pneumococcal infections, COVID-19, and other vaccines (e.g., against hepatitis B, tetanus, and tick-borne encephalitis). Additionally, respondents were asked whether they had experienced any adverse events following immunization (AEFI), commonly referred to as adverse vaccine reactions (AVRs). Adverse events were defined broadly to include symptoms such as localized pain or redness, fever, muscle aches, malaise, joint pain, or swelling. Self-reported data on vaccination status and adverse reactions formed the basis for the subsequent analysis.

The participants answered questions specifically regarding their vaccination history over the past 3 years. The survey captured information about vaccinations against influenza, pneumococcal infections, COVID-19, and other vaccines (e.g., against hepatitis B, tetanus, and tick-borne encephalitis). Additionally, respondents were asked whether they had experienced any adverse events following immunization (AEFI), commonly referred to as adverse vaccine reactions (AVRs). Adverse events were defined broadly to include symptoms such as localized pain or redness, fever, muscle aches, malaise, joint pain, or swelling. Self-reported data on vaccination status and adverse reactions formed the basis for the subsequent analysis. Participants who reported adverse events were asked to describe their symptoms in an open-ended format. These descriptions were later reviewed by the research team and categorized into one of three groups: (1) injection site reactions (e.g., pain, redness, swelling), (2) systemic symptoms (e.g., fever, headache, fatigue, muscle or joint pain), or (3) unclassifiable descriptions (e.g., vague or inconsistent responses). The classification was performed independently by two researchers, with discrepancies resolved through discussion. The questionnaire included both closed- and open-ended items addressing sociodemographic characteristics, health status, vaccination uptake, and adverse reactions. It was specifically designed for this study and pre-tested informally for clarity and comprehensibility among a small group of older adults before full deployment.

### 2.3. Statistical Analysis

All statistical analyses were conducted using Statistica 10.0 (StatSoft Polska Sp. z o.o., Kraków, Poland) and PQStat (PQStat Software 1.8.6.102, Poznań, Poland). Descriptive statistics for continuous variables were presented as mean (M), standard deviation (SD), median (Me), minimum (Min), maximum (Max), and the 25th (Q25) and 75th (Q75) percentiles. Categorical variables were described using frequencies (N) and percentages (%). Comparisons between groups (e.g., by gender) were made using the chi-square (χ^2^) test for categorical variables and the independent samples *t*-test for continuous variables. Relationships between continuous variables were assessed using Pearson’s correlation coefficient (r). A significance threshold was set at *p* < 0.05.

## 3. Results

A total of 2040 individuals participated in the study, with women comprising the majority (68.9%). The mean age of the respondents was 65.4 years (SD = 8.29). The largest age group included individuals aged 60–75 years (52.6%). The independent samples *t*-test revealed statistically significant differences in mean age between women and men (t = −5.246, *p* < 0.001), with men being, on average, older (66.8 years) than women (64.8 years). The variance test (F = 1.086, *p* = 0.230) showed no statistically significant differences in the standard deviations of age between genders. Table 1 presents the basic demographic characteristics of the study participants, including gender, age, and distribution across age groups.

Among all respondents who answered the question regarding vaccinations received in the past 3 years, the most frequently reported was vaccination against COVID-19 (86.9%). Influenza vaccination was declared by 45.5% of participants, and pneumococcal vaccination by 15.1%. Other vaccinations, including immunizations against hepatitis, measles, meningococcal infections, jaundice, tetanus, and tick-borne encephalitis, were reported by only 0.8% of respondents. Statistical analysis revealed significant differences between women and men in the uptake of influenza vaccination (*p* < 0.05), while no statistically significant differences were observed regarding pneumococcal and COVID-19 vaccinations (*p* > 0.05). Detailed data are presented in Table 2.

Among the study participants, 13.9% reported adverse events following COVID-19 vaccination. Symptoms occurred significantly more frequently in women (16.1%) compared to men (8.8%) (*p* < 0.001). In the case of other vaccinations, adverse events were reported by 2.0% of respondents, with no statistically significant differences between genders (*p* = 0.199). The most commonly reported symptoms included headache, pain, and redness at the injection site, malaise, muscle pain, joint pain, weakness, and elevated body temperature. Data regarding the occurrence of adverse events following immunization are presented in Table 3. Among 270 participants who reported adverse events following COVID-19 vaccination, 43 individuals (15.9%) experienced only injection site reactions, such as pain, redness, or swelling. A total of 108 participants (40.0%) reported only systemic symptoms, including fever, headache, muscle aches, or fatigue. Mixed symptoms, involving both injection site reactions and systemic symptoms, were reported by 113 individuals (41.9%). In six cases (2.2%), adverse events were classified as serious, and in another six cases (2.2%), no specific symptom data were available. Among 39 participants who reported adverse events following vaccinations other than for COVID-19, 14 individuals (35.9%) experienced systemic symptoms such as fever, malaise, or muscle aches. Injection site reactions, including pain, redness, or swelling, were reported by seven individuals (17.9%). Three participants (7.7%) experienced both types of symptoms. In 15 cases (38.5%), symptom descriptions were not specific enough to allow for classification.

To compare the mean number of vaccinations and the mean frequency of adverse events following immunization between genders, the independent samples *t*-test was applied. Statistically significant differences between women and men were found in the number of vaccinations received over the past 3 years and in the frequency of adverse events following COVID-19 vaccination (*p* < 0.001). Detailed results are presented in Table 4.

To assess the relationship between the number of vaccinations, COVID-19 vaccination, and the occurrence of adverse events following immunization, Pearson’s correlation analysis was applied. A statistically significant, although very weak, negative correlation was found between the number of vaccinations received over the past 3 years and the age of the respondents (r = −0.088; *p* = 0.001). No significant relationship was observed between age and the decision to receive the COVID-19 vaccination (*p* = 0.657). However, a weak positive correlation was identified between age and the occurrence of adverse events following COVID-19 vaccination (r = 0.175; *p* < 0.001), as well as following other vaccinations (r = 0.067; *p* = 0.011) (Table 5).

## 4. Discussion

The findings of this population-based study reveal important patterns in vaccination coverage and adverse events among older adults in Płock, Poland, with implications for public health strategies both locally and internationally. The high COVID-19 vaccination rate (86.9%) observed in our cohort significantly surpasses the coverage rates for influenza (45.5%) and pneumococcal vaccines (15.1%), highlighting a notable disparity in immunization patterns that merits attention from policymakers and healthcare providers. The high COVID-19 vaccination coverage in Płock may also be partly attributed to proactive municipal health outreach initiatives, such as mobile vaccination units, partnerships with local senior organizations (e.g., Universities of the Third Age), and targeted information campaigns disseminated via community health centers. These efforts were part of a coordinated regional response to the pandemic and may have enhanced accessibility and trust among older residents. However, such structured outreach has not been systematically extended to other adult immunization programs, which could explain the lower uptake for influenza and pneumococcal vaccines.

The exceptional uptake of COVID-19 vaccination likely reflects the heightened public awareness and comprehensive mobilization efforts during the pandemic, which succeeded in reaching a substantial proportion of older adults. This finding aligns with recent research by Machado et al. (2023), who documented similarly high COVID-19 vaccination rates among older adults across several European countries during the pandemic period [35]. However, the substantially lower coverage for influenza and pneumococcal vaccines indicates persistent challenges in achieving comprehensive protection against common respiratory pathogens in this vulnerable population. Barrat et. al. noted comparable disparities in their analysis of vaccination patterns among older adults in multiple high-income countries, suggesting this is a widespread phenomenon requiring a coordinated international response [36].

The observed gender differences in vaccination patterns and adverse event reporting are particularly noteworthy. Men in our study demonstrated higher influenza vaccination rates compared to women (50.7% vs. 43.4%, *p* < 0.05), contradicting the traditionally observed pattern of greater preventive health behaviors among women. This unexpected finding warrants further investigation and may reflect unique sociocultural factors or targeted outreach efforts in the study region. Interestingly, men in our sample reported higher influenza vaccination rates compared to women. This may reflect gendered health behaviors, where older men are more likely to comply with physician recommendations, possibly due to a greater perceived risk of complications. A systematic review by Zintel et al. found that men across multiple countries exhibited higher COVID-19 vaccine acceptance, while women more frequently expressed concerns about vaccine safety and adverse effects [37]. Delpech et al. further showed that older women tend to seek more detailed explanations and emotional reassurance before accepting vaccination, in contrast to men’s more passive reliance on medical authority [38]. These psychosocial dynamics suggest that vaccination campaigns targeting older adults should incorporate both rational and emotionally attuned messages, especially when addressing women’s specific concerns. Conversely, women reported significantly more adverse events following COVID-19 vaccination than men (16.1% vs. 8.8%, *p* < 0.001), which aligns with emerging evidence on sex-based differences in vaccine reactogenicity. Other researchers documented similar gender disparities in adverse event reporting following COVID-19 vaccination, attributing these differences to both biological factors and gender-specific reporting behaviors [39,40,41]. Emerging immunological research by Morgan et al. (2022) suggests that estrogen-related immune responses may contribute to heightened reactogenicity among females across multiple vaccine types [42].

The weak negative correlation between age and number of vaccinations received (r = −0.088, *p* = 0.001) suggests that the oldest members of our cohort may face unique barriers to accessing multiple vaccines, which requires focused attention. This finding parallels research by Raciborski et al. (2021), who identified decreasing vaccination rates with advancing age among Polish seniors, particularly among those over 75 years [43]. However, the observed positive correlation between age and reporting of adverse events following COVID-19 vaccination (r = 0.175, *p* < 0.001) contrasts with some international data. While Yaamika et al. (2023) found that older adults typically report fewer adverse events than younger populations in their multinational analysis, our findings suggest that age-related vulnerability may manifest differently in specific populations [44]. This discrepancy highlights the importance of context-specific research and surveillance.

The overall low incidence of adverse events reported after COVID-19 vaccination (13.9%) and other vaccines (2.0%) in our population is reassuring and consistent with international safety monitoring data. A comprehensive meta-analysis by Xu et al. documented similarly low rates of adverse events among older adults across multiple countries, confirming the favorable safety profile of COVID-19 vaccines in this demographic [45]. The predominantly mild nature of reported symptoms in our study further reinforces the positive benefit–risk balance of vaccination in older adults, as emphasized by Soiza et al. (2021) in their systematic review of COVID-19 vaccination outcomes in geriatric populations [46].

Our findings have important implications for public health practice and policy. The disparity between COVID-19 vaccination rates and those for influenza and pneumococcal vaccines suggests an opportunity to leverage the infrastructure and heightened awareness established during the pandemic to improve coverage of other recommended immunizations. Targeted communication strategies addressing gender-specific concerns and barriers may help reduce disparities in both vaccine uptake and adverse event reporting [47]. Additionally, the correlation between age and vaccination patterns indicates a need for age-tailored approaches to reach the oldest segments of the population effectively.

Future research should employ mixed-methods approaches to explore the underlying determinants of gender and age differences in vaccination behaviors and adverse event reporting. Longitudinal studies tracking vaccination patterns over time would provide valuable insights into the sustainability of COVID-19 vaccination behaviors and their potential spillover effects on other recommended vaccines. Implementation research evaluating tailored interventions to increase influenza and pneumococcal vaccination among older adults is also warranted, as suggested by recent work from Nowak et al. on effective strategies to improve vaccination rates in older populations [48].

### Study Limitation

This study has certain limitations. Firstly, the data were collected through self-reported questionnaires, which, despite being structured and standardized, may still be subject to reporting bias. Participants might have under- or over-reported their vaccination history or adverse event experiences based on subjective perception, memory limitations, or personal interpretation. Secondly, although the study included a large and demographically reflective sample of older adults from one urban area, it focused solely on residents of Płock. Therefore, the results cannot be directly generalized to populations living in other regions of Poland, particularly rural areas, where healthcare access and vaccination attitudes may differ. Thirdly, there was a gender imbalance among participants, with women constituting the majority of the sample. While this reflects demographic trends in aging populations, especially in Poland, it may nonetheless influence the interpretation of gender-related findings and should be considered in future research. Despite these limitations, the study offers valuable insights into vaccination behaviors and adverse event experiences among older adults and can serve as a foundation for broader, possibly longitudinal, investigations across different demographic and geographic contexts. Fourthly, the study did not include information on organizational aspects of vaccination, such as the setting in which vaccinations were performed, the medical professionals involved in qualification and administration, or the methods used to inform and invite participants. Likewise, communication between healthcare providers and patients regarding potential adverse events was not assessed. These system-level factors, although standardized under Polish vaccination protocols, may play a significant role in shaping individuals’ decisions and perceptions. Future studies should consider including such variables to provide a more comprehensive understanding of vaccine uptake dynamics.

## 5. Conclusions

A high rate of COVID-19 vaccination was observed among seniors, with lower uptake for influenza and pneumococcal vaccines. Women reported adverse events following COVID-19 vaccination more frequently than men. Age showed a weak correlation with vaccination behaviors and the occurrence of adverse events. These findings indicate the need to strengthen vaccination programs targeting infections other than COVID-19 and to develop tailored communication strategies considering gender differences. Moreover, public health efforts should build on the organizational and informational infrastructure established during the COVID-19 pandemic to promote broader immunization coverage. Special attention should be paid to enhancing influenza and pneumococcal vaccination uptake among older adults. Gender-sensitive educational campaigns and age-adapted outreach initiatives may help address both hesitancy and under-vaccination in specific subgroups. Future vaccination campaigns targeting older adults should leverage trusted healthcare providers, simplify access (e.g., on-site vaccinations at primary care clinics or community centers), and address gender-specific concerns to reduce hesitancy and improve overall vaccine coverage.

## Figures and Tables

**Table 1:** Basic demographic characteristics of the study participants.

Variable	Category	N	%	Mean Age	SD	Min	Max	Med.	Q25	Q75	*p*
Sex	Female	1406	68.90%	64.8	8.334	55	100	64	57	70	<0.001
	Male	634	31.10%	66.8	7.998	55	93	67	60	72	
	Total	2040	100.00%	65.4	8.285	55	100	65	58	70	
Age group	≤60 years	664	32.50%								
	60–75 years	1073	52.60%								
	75–90 years	279	13.70%								
	>90 years	24	1.20%								

**Table 2:** Frequency of vaccinations in the past 3 years by gender.

Type of Vaccination	Women—n (%)	Men—n (%)	Total—n (%)	*p*
Influenza	512 (43.4%)	241 (50.7%)	753 (45.5%)	<0.05
Pneumococcal	184 (15.6%)	66 (13.9%)	250 (15.1%)	>0.05
COVID-19	1195 (87.4%)	537 (85.6%)	1732 (86.9%)	>0.05
Other	4 (0.3%)	7 (1.1%)	11 (0.8%)	-

**Table 3:** Occurrence of adverse events following immunization (AEFI) by gender.

Type of Vaccination	Women–n (%)	Men—n (%)	Total—n (%)	*p*
AEFI after COVID-19 vaccination	215 (16.1%)	55 (8.8%)	270 (13.9%)	<0.001
AEFI after other vaccinations	31 (2.2%)	8 (1.3%)	39 (2.0%)	0.199

**Table 4:** Differences between women and men in the number of vaccinations and adverse events following immunization.

Parameter	Vaccinations in the Past 3 Years	COVID-19 Vaccination	AEFI After COVID-19 Vaccination	AEFI After Other Vaccinations
Mean–women	2.479	1.129	1.839	1.977
Mean–men	2.141	1.137	1.912	1.987
*t*-value	4.192	−0.525	−4.441	−1.529
df	1439	2038	2038	2038
*p*	<0.001	0.6	<0.001	0.126
SD–women	1.447	0.335	0.368	0.149
SD–men	1.299	0.344	0.284	0.112
F variance ratio	1.242	1.056	1.68	1.784
*p* variance test	0.009	0.411	<0.001	<0.001

Abbreviations: AEFI—adverse events following immunization; SD—standard deviation; *t*—*t*-value (Student’s *t*-test); df—degrees of freedom; F—F-statistic (variance ratio); *p*—probability value (significance level).

**Table 5:** Correlations between age and vaccination-related outcomes.

Zagadnienie	r	*p*
Vaccinations in the past 3 years	−0.088	0.001
Received COVID-19 vaccination	0.012	0.657
Adverse events after COVID-19 vaccination	0.175	0
Adverse events after other vaccinations	0.067	0.011

## Data Availability

Data are available upon reasonable request.

## Abbreviations

The following abbreviations are used in this manuscript:

AEFI	Adverse Events Following Immunization
CDC	Centers for Disease Control and Prevention
COVID-19	Coronavirus Disease 2019
ECDC	European Centre for Disease Prevention and Control
IAGG	International Association of Gerontology and Geriatrics
IFA	International Federation on Ageing
MMSE	Mini Mental State Examination
NOP	Niepożądany Odczyn Poszczepienny (Adverse Post-Vaccination Reaction)
WHO	World Health Organization
PQStat	Polish Statistical Software for Data Analysis
U3A	University of the Third Age
